# Resistance evaluation and surveillance initiative for schistosomiasis treatment: study protocol for the RESIST project

**DOI:** 10.1186/s12879-025-12474-1

**Published:** 2026-01-08

**Authors:** Stefanie Knopp, Said M. Ali, Norbert van Dijk, Aidan M. Emery, Tom Pennance, Bonnie L. Webster, Luc E. Coffeng

**Affiliations:** 1 Swiss Tropical and Public Health Institute, Kreuzstrasse 2, Allschwil, 4123 Switzerland; 2 University of Basel, Petersplatz 1, Basel, 4002 Switzerland; 3 Public Health Laboratory - Ivo de Carneri, P.O. Box 122, Wawi, Chake Chake, Pemba, United Republic of Tanzania; 4 Department of Public Health, Erasmus MC, University Medical Center Rotterdam, P.O. Box 2040, Rotterdam, 3000 CA The Netherlands; 5 Natural History Museum, Cromwell Road, London, SW7 5BD UK

**Keywords:** Elimination, Genomics, Hotspot, Interruption of transmission, Mass drug administration, Modelling, Praziquantel, Resistance, Schistosomiasis, S. haematobium, Treatment, Zanzibar

## Abstract

**Background:**

The Zanzibar islands have implemented mass drug administration (MDA) with praziquantel against *Schistosoma haematobium* infections since the 2000s, achieving elimination of schistosomiasis as a public health problem in most areas. However, a few hotspots with high prevalence of infection remain, where MDA-driven selection of drug-resistance within the *S. haematobium* populations might contribute to persistent transmission. Recent advances in understanding the molecular basis of praziquantel action on the *Schistosoma* transient receptor potential ion channel (TRPM_PZQ_) now facilitate screening *Schistosoma* populations for genetic signatures that may point to reduced praziquantel efficacy, or even resistance.

**Methods:**

This observational study is implemented from 2024 to 2027 and contains four components: (1) Cross-sectional surveys in Pemba: Single urine samples are collected from ~ 14,400 students across 15 schools to assess the geographic distribution of *S. haematobium* infection prevalence and intensity, and for miracidia collection. (2) Longitudinal studies in Pemba: Quintuple urine samples are collected from ~ 945 students across two schools before and two weeks after MDA to assess *S. haematobium* egg reduction rates (ERR), and for miracidia collection. (3) Genome-wide analyses of the *S. haematobium* miracidia collected in 1 and 2, (a) across Pemba over multiple time points (including archived specimens collected in 2012 and 2017) to quantify spatiotemporal patterns of parasite genetic variation; and (b) before and two weeks after MDA to identify any genetic variants under selection and potentially associated with praziquantel sensitivity. 4) Modelling to: (a) analyse correlation of observed *S. haematobium* ERR and miracidia genetic profiles; (b) predict the impact of MDA and mitigation strategies on emergence of drug resistance; and (c) design cost-efficient survey strategies for pharmacovigilance.

**Discussion:**

The combination of parasitological fieldwork, genomic analyses, and mathematical modelling will deliver new insights into how MDA has shaped genetic diversity of *S. haematobium* populations, how this might affect drug efficacy and contribute to persistent hotspots, and how this can best be monitored and mitigated. This project will lay the foundations for population-based research into drug resistance in *Schistosoma* with important implications for novel drugs in the pipeline.

**Trial registration:**

ISRCTN, ISRCTN59331501. Registered 24 October 2024, https://www.isrctn.com/ISRCTN59331501.

## Background

Schistosomiasis is a neglected tropical disease, which affects millions of people in 78 endemic countries [1, 2]. Regular mass drug administration (MDA) is the main strategy for schistosomiasis control [1, 3]. Over the past two decades, many endemic countries have successfully reduced schistosomiasis, most likely through the scale-up of MDA [4]. The World Health Organization (WHO) has set the goals to eliminate schistosomiasis as public health problem globally and to interrupt transmission in selected areas by 2030 [1].

The RESIST project will identify if praziquantel (PZQ) resistance is an endgame challenge for the elimination of urogenital schistosomiasis, which is caused by *Schistosoma haematobium*, and if resistance is found, how it may be monitored and mitigated. It will be implemented on Pemba island, which is part of the Zanzibar archipelago of the United Republic of Tanzania. The Zanzibar Ministry of Health has an impressive track record in conducting large-scale treatment programmes against *S. haematobium* infections since the 1980s [5, 6]. Regular MDA of PZQ to schoolchildren started in the early 2000s [5]. From 2013 to 2016, biannual MDA was conducted in schools and communities within the efforts of the Zanzibar Elimination of Schistosomiasis Transmission (ZEST) project [7]. Since then, with a gap in 2019, MDA has been implemented in schools and communities annually, and even biannually in 2023. From 2017 onward, elimination of *S. haematobium* as a public health problem was achieved, i.e. overall prevalence of heavy intensity infections < 1% [7, 8]. However, across the islands, there is marked spatial heterogeneity in infection levels with many areas that show a very low prevalence (≤ 1%) but a few areas where prevalence in schoolchildren persisted at 5–10% in 2020, despite multiple rounds of MDA, which were labelled as “hotspots” [9].

The occurrence of these hotspots of persistent transmission may be explained by several factors including rurality, proximity to water bodies that contain snail intermediate hosts, and economic standard of households clustered in certain areas [10, 11]. However, given the two decades of intense PZQ administration to the population of Zanzibar, it is possible that hotspots are driven by a reduced impact of PZQ on the *S. haematobium* populations, due to drug resistance [12–14]. Given the lack of treatment alternatives for schistosomiasis, potential resistance against PZQ would have far-reaching implications for sustained control and interruption of transmission, both in Zanzibar and elsewhere, as well as substantially increasing the urgency for novel therapeutics, despite the current scarcity in the development pipeline [13].

This project proposes to investigate PZQ resistance as a potential cause of *S. haematobium* transmission hotspots in Pemba, leveraging a unique opportunity that has arisen due to: (a) the discovery of the *Schistosoma* molecular target of PZQ action in a transient receptor potential ion channel (TRPM_PZQ_) in 2019 together with the identification of specific genetic mutations, such as the missense mutation in *Sm*TRPM_PZQ_[N1388T] that can cause a complete lack of response (i.e. resistance) of *Sm*TRPM_PZQ_ to PZQ compared with the wild-type [15–20]; (b) robust, long-term monitoring of the changing schistosomiasis epidemiology in Zanzibar via annual surveys of school-aged children and adults from 2012 to 2021 [7–9]; (c) an available historical collection of Zanzibari *S. haematobium* samples stored in the Schistosomiasis Collection at the Natural History Museum (SCAN), London, United Kingdom [21]; and (d) the recent development of quantitative frameworks to predict trends in drug resistance in human helminth infections and design cost-efficient survey strategies for pharmacovigilance [14, 22–24].

With a combination of parasitological fieldwork, genomic analyses, and mathematical modelling, the RESIST project aims to deliver new insights into how MDA has shaped the genetic diversity of *S. haematobium* over space and time in Zanzibar, how this has potentially affected drug efficacy and contributed to the persistence of hotspots, and how emerging resistance can best be monitored and mitigated.

## Methods/Design

### Study aim

The overall goal of this project is to understand whether PZQ resistance has contributed to the existence of persistent *S. haematobium* hotspots in Pemba, and if so, how to address this.

### Primary and secondary objectives

The primary objective of this study is to genetically profile *S. haematobium* miracidia from different locations in Pemba in 2024/25 and identify any genetic markers associated with PZQ resistance.

Secondary objectives are:

1. To assess the prevalence and intensity of infections (*S. haematobium* egg counts) in students from 17 schools in 2024/25;
2. To assess the prevalence and intensity of infections (*S. haematobium* egg counts) in students from two schools before and after MDA in 2024/25;
3. To genetically profile *S. haematobium* miracidia from 17 different schools in Pemba collected in 2024/25;
4. To genetically profile *S. haematobium* miracidia from the same 17 schools in Pemba collected in 2012 and 2017;
5. To genetically profile *S. haematobium* miracidia collected before and after MDA from two different schools in Pemba in 2024/25;
6. To quantify spatiotemporal genetic diversity patterns of *S. haematobium* populations;
7. To identify allele frequency changes/shifts over space and time;
8. To investigate the potential association between genetic variants and PZQ sensitivity based on *S. haematobium* egg counts;
9. To investigate how novel *S. haematobium* TRPM_PZQ_ genetic variants alter PZQ sensitivity phenotypes based on functional profiling of TRPM_PZQ_ mutations in cell lines;
10. To assess the effectiveness of PZQ on clearing *S. haematobium* infections at the individual level and also the entire population involved in PZQ MDA programmes;
11. To predict the impact of PZQ MDA and resistance mitigation strategies on population dynamics of drug resistance in *S. haematobium*;
12. To design cost-efficient survey strategies for pharmacovigilance.

### Study design

The fieldwork for the RESIST project is conducted on Pemba, an island of the Zanzibar archipelago, United Republic of Tanzania. It is designed as an observational study to inform mathematical models and entails the following components:

1. Parasitological surveys:
  - Cross-sectional surveys: Collect single urine samples from students in 15 schools that were also part of the ZEST study (2012–2017), to assess the geographic distribution of *S. haematobium* prevalence and intensity of infection, and to obtain *S. haematobium* miracidia isolated from eggs excreted by infected individuals.
  - Longitudinal studies: Collect quintuple urine samples (1/day over five different days) from students in two schools before and two weeks after PZQ MDA to assess *S. haematobium* egg reduction rates (ERR), estimate PZQ drug efficacy and effectiveness of MDA, and to obtain *S. haematobium* miracidia isolated from eggs excreted by infected individuals.
2. Genomic analyses of isolated and preserved *S. haematobium* miracidia, collected from infected individuals:
  - from across Pemba over multiple time points (2012 and 2017 from the SCAN archives, and 2024 and 2025 from this study) to quantify spatiotemporal patterns in *S. haematobium* genetic variation;
  - before and two weeks after PZQ MDA to screen for TRPM_PZQ_ variants known to be associated with PZQ resistance [15, 19, 25, 26], and to identify any additional genomic markers whose frequency increases post-MDA, indicating potential selection in the surviving *S. haematobium* population.
3. Mathematical modelling, including:
  - Correlation of observed individual- and population-level *S. haematobium* ERR with genetic signatures and estimation of underlying worm-level PZQ efficacy to inform modelling;
  - Prediction of impact of MDA with PZQ and mitigation strategies on population dynamics of drug resistance in *S. haematobium*, using the data from objectives 1 and 2;
  - Design of cost-efficient survey strategies for pharmacovigilance.

### Study setting

**Fig. 1:**
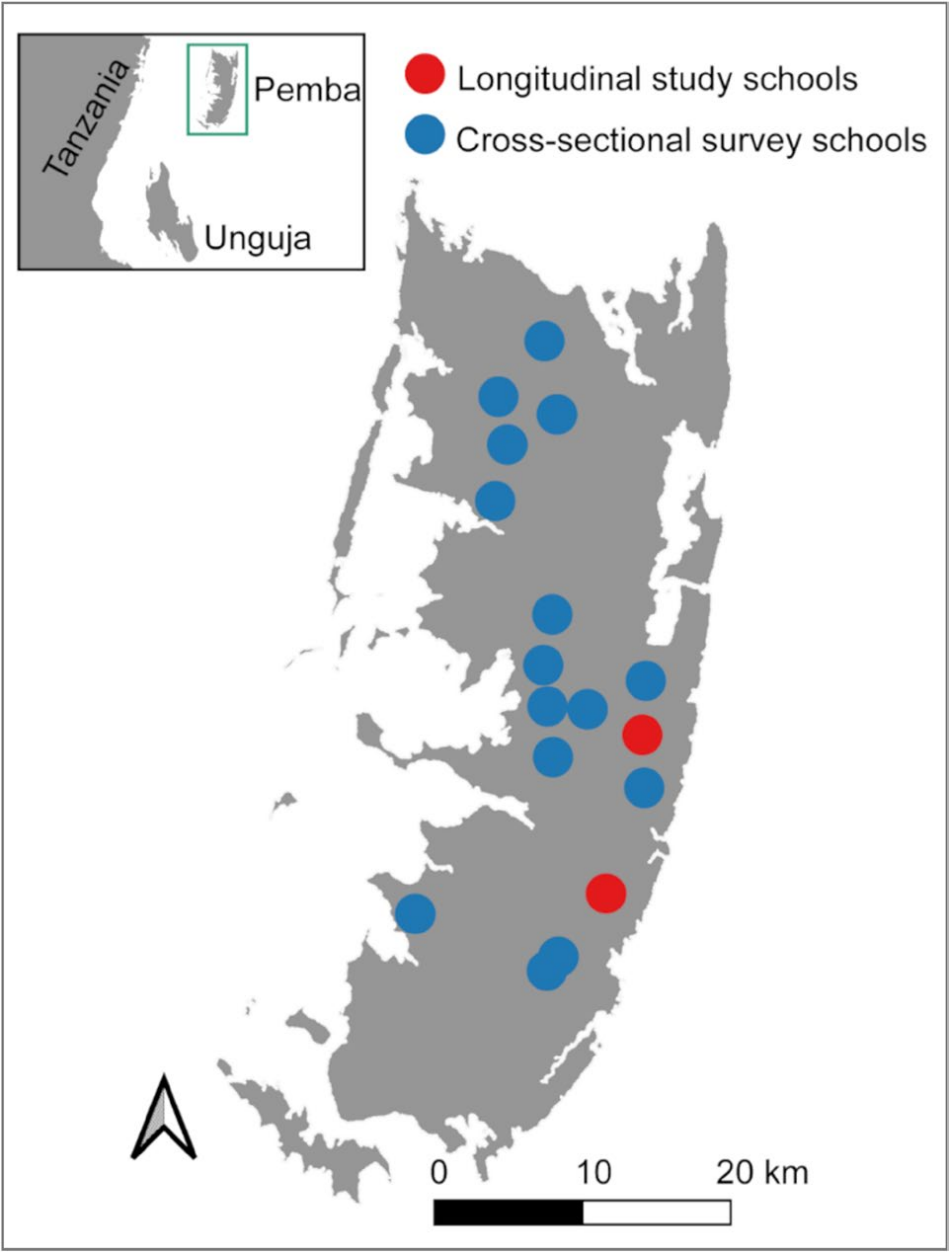
Map of Pemba, United Republic of Tanzania, showing the location of the 17 study schools. Inset map shows position of Pemba island (green box) situated in the Indian Ocean relative to mainland Tanzania and Unguja island. The image base map (United Republic of Tanzania – Administrative areas) was downloaded from DIVA-GIS (diva-gis.org). The data are published under the GNU General Public License. Maps were produced in QGIS v3.44.5

The RESIST project includes partners from the Public Health Laboratory-Ivo de Carneri (PHL-IdC), Pemba, United Republic of Tanzania; Swiss Tropical and Public Health Institute (Swiss TPH), Allschwil, Switzerland; Natural History Museum (NHM), London, United Kingdom; and Erasmus University Medical Center (Erasmus MC), Rotterdam, the Netherlands. The fieldwork for the RESIST project is conducted on Pemba, the northernmost island of the Zanzibar archipelago. The study locations are 17 public primary schools (Fig. 1), which were selected among 45 schools that were part of the ZEST project in Pemba from 2012 to 2017 [27]. Public primary schools in Zanzibar include grades 1–7, with ages typically ranging from 5 to 16 years.

### Characteristics of participants

#### Participant eligibility

Individuals fulfilling all the following inclusion criteria are eligible for participation:

- Attendance of grades 1–7 in one among the 17 schools selected to be part of the study.
- Aged 5–16 years.
- Randomised to participate in study (if the number of attending children is greater than required).
- Written informed consent signed by the parents.
- Written assent signed by the participant if aged 12–16 years old.

The presence of any one of the following exclusion criteria will lead to the exclusion from participation:

- Not attending any of the 17 selected schools.
- Not attending grades 1–7.
- Not aged 5–16 years.
- Not randomised to participate in the study (if the number of attending children is greater than required).
- No written informed consent signed by the parents submitted.
- No written assent signed by the participant if aged 12–16 years old.
- Observed or reported clinically significant severe disease.

#### Participant recruitment and urine sample collection

Before the onset of the study, we will conduct information meetings with members of the Zanzibar Ministry of Health and district authorities, and local shehas (community leaders) and principals of the 17 schools where the study will be conducted to explain the purpose and procedures of the study. The shehas will be invited to inform their communities, and the school principals to inform their teachers about the forthcoming study.

Participants for this project will be recruited from 17 schools via two study designs:

##### Cross-sectional surveys in 15 primary schools before PZQ MDA

Cross-sectional surveys will be conducted in 15 primary schools before PZQ MDA. On Day 1, the study aims and procedures will be explained to the children. If the school size is below 1200 registered students, all students will be included. If the school size exceeds 1200 registered students, children will be randomly selected to participate in the cross-sectional surveys. For randomization, in a first step, classes A, B, C or D from each grade will be selected based on a randomized list. Subsequently, in each randomly selected class per grades 1–7, all children will line up, stratified by gender. Subsequently, we will systematically select each third child in the lines to be included in the study until 50 children per class are reached. This procedure will be continued until we reach a total of 1200 selected children from seven grades per school. Selected children will be registered and receive a personal participant ID code. They will be given an informed consent form (ICF) to give to their caregivers. On Day 2, children who submit the ICF signed by their caregivers, and additionally an assent form with their own signature if they are aged 12–16 years, will be asked about their demographic information, place of residency, recent travel history outside Pemba, and last PZQ treatment for schistosomiasis. This information will be recorded in a questionnaire with Open Data Kit Software (ODK) on tablet computers (Samsung Galaxy Tab A 2019). After the interview, each child will be given a urine container (100 ml) labelled with the unique participant ID code and asked to fill the container with their own urine sample between 10 am and 3 pm, which is then submitted to the field enumerators. In total, from the 15 schools, we will enrol a maximum of 18,000 students. Accounting for a 20% drop-out due to non-consenting parents, absenteeism of children or inability to produce a urine sample of sufficient volume, we aim for a final sample size of 960 students per school and 14,400 in total.

##### Longitudinal study in two primary schools before and after MDA

A longitudinal study of repeated parasitological surveys of children (before and after PZQ MDA) will be conducted in two primary schools with known persistent 5–10% prevalence of *S. haematobium* infection. A few days before MDA, on Day 1, the study aims and procedures will be explained to the children. Subsequently, children will be randomly selected to participate in the longitudinal study. The random selection will be conducted as described for the cross-sectional surveys and until 50 children per class are selected. This procedure will be continued until we reach a total of 800 selected children from seven grades per school. Selected children will be registered and receive a personal participant ID code. They will be given an ICF to give to their caregivers. Parents and caregivers will be invited to an information meeting in the school on the same evening, where they will have the chance to ask questions and discuss concerns about the study. The questionnaire interview and sample collection on Day 2 is similar to the procedure described for the cross-sectional surveys. In addition, in this longitudinal study, the participating children will be asked to produce a urine sample also on Days 3–6, for a total of five (1/day) urines provided on five different days per person before MDA. Next, PZQ MDA will be implemented by qualified staff of the NTD Unit of the Zanzibar Ministry of Health in the two schools, aligned with the RESIST project and in consultation and collaboration with the RESIST study team. During the PZQ MDA, the RESIST study team will record whether or not a child participates in the MDA. If they participate, the number of PZQ tablets each treated study participant receives and takes, and whether they vomit immediately after treatment, will be recorded. Two weeks after MDA, the sampling procedures from Day 2–6 will be repeated, targeting the same previously selected children. In total, we will enrol 1600 students from the two schools. Accounting for a daily drop-out rate of ~ 10%, we expect to receive five urine samples before and after MDA from a total of 945 participants.

### Treatment

In the cross-sectional surveys involving 15 schools, students will be treated with PZQ (40 mg/kg) during the MDA that will be conducted in each school immediately after the conclusion of the survey in their school. In the longitudinal study in two schools, after the first survey, all students will be offered PZQ via the MDA conducted by the Zanzibar NTD Unit in collaboration with the RESIST study team. Students that are identified as still infected with *S. haematobium* post MDA will be re-treated with PZQ (40 mg/kg) by a qualified member of the Zanzibar NTD Unit in collaboration with the study team once the second survey in their school is concluded. All students participating in the MDA will be offered a porridge meal before PZQ treatment. Students that are re-treated after MDA will receive biscuits before treatment. PZQ will be administered as a single oral dose (40 mg/kg), using a validated dose pole that is based on height rather than weight [28]. Children will be offered tap water to help with swallowing the tablets.

### Laboratory examinations

#### Urine filtration microscopy for *S. haematobium* egg detection

Urine samples will be collected in individual sample pots from each participant and labelled with the participant ID code. The urine samples will be transferred from the school to the PHL-IdC on the day of collection.

In the laboratory, after mixing, 10 ml of each urine sample will be taken up with a 10 ml plastic syringe and filtered through a 20 micron 13 mm filter (Sefar Nitex 03–25/19, United Kingdom) held within a Swinnex filter holder (Merck, Germany) attached to the syringe to catch any *S. haematobium* eggs. Each individual filter will be mounted on a separate microscope slide, labelled with the participant ID code, and covered with a piece of hydrophilic cellophane soaked in glycerol. A few drops of 10% Lugol’s iodine solution will be added on top of the cellophane to stain any *S. haematobium* eggs. The filter area will be examined with a light microscope for the presence and number of *S. haematobium* eggs by experienced laboratory technicians. The presence and number of *S. haematobium* eggs will be recorded in the case report form (CRF). All *S. haematobium*-positive urine samples will be subjected to miracidia hatching as described below.

#### Collection of *S. haematobium* miracidia

Each urine sample identified as *S. haematobium* egg-positive that has urine remaining after urine filtration microscopy (i.e. supplied volume of urine from participant was > 10 ml) will be subjected to miracidia isolation. For this purpose, urine will be mixed vigorously, using a 10 ml plastic syringe. The whole volume of the remaining urine (up to 90 ml) of each sample will be filtered through a single 20 micron 13 mm filter as described above, or through multiple filters used for the same sample if blockage occurred. For each urine sample, the filter(s) will then be placed in a petri dish labelled with the participant ID and containing room temperature distilled water. The petri dish containing the urine filter will be placed in indirect sunlight to facilitate miracidial hatching. Miracidia will be visualized under a binocular dissection microscope and individually captured using a p20 micropipette and 20 µl ultra point tip in 3 µl of clean water. Miracidia will be then pipetted individually either onto a QIAcard FTA™ Indicating Classic cards (Qiagen, Hilden, Germany) and left to dry, or into individual 0.2 ml strip tubes containing 9 µl of DNA/RNA Shield (Zymo Research, California, United States of America) and frozen at -20 °C. Both methods preserve miracidia DNA for genetic analyses. Up to 50 miracidia will be collected from each participant for genetic analyses using established methods [29].

Only biological material of *S. haematobium* origin will be captured and only *S. haematobium* DNA will be analysed.

Each FTA card will be labelled with the participant ID, date of collection, number of miracidia, and name of the technician involved in the collecting. Miracidia in 0.2 ml strip tubes of DNA/RNA shield will be accessioned with unique numbers connected back to the sample metadata. A corresponding miracidia collection form and spreadsheet will also be filled out containing equivalent information, copied, and retained in a separate location to the samples.

#### Genomic analysis of *S. haematobium* miracidia

To explore potential links between the genetics of *S. haematobium* populations and variation in PZQ efficacy (based on ERRs), we will perform genome wide analyses of individual *S. haematobium* miracidia collected from infected individuals.

We aim to analyse *S. haematobium* miracidia (*n* = 1260) from ~ 420 individuals (three miracidia per person) pre-MDA across all 17 schools. In the two schools that are part of the longitudinal studies, we will also analyse all *S. haematobium* miracidia collected from individuals that are found to be egg-positive post-MDA.

Individual miracidia will be analysed by adapting established whole genome sequencing (WGS) methodologies and bioinformatic pipelines/tools [17, 25, 30, 31]. A WGS approach will be used so that both, pre-defined targets (i.e. TRPM_PZQ_) and broader genome-wide signals can be investigated, allowing:

1. The first whole genome-level characterisation of natural *S. haematobium* populations in Pemba, together with the first investigation of the *S. haematobium* TRPM_PZQ_ channel (previous research has focused on TRPM_PZQ_ channel variation in *S. mansoni* populations elsewhere in sub-Saharan Africa);
2. Inter- and intra-population structure analyses to infer the variation and ancestry of *S. haematobium* across Pemba, together with identification of genomic regions showing putative evidence for positive selection following MDA, which may indicate parasite genetic adaptation/differentiation relevant to PZQ pressure.

**Fig. 2:**
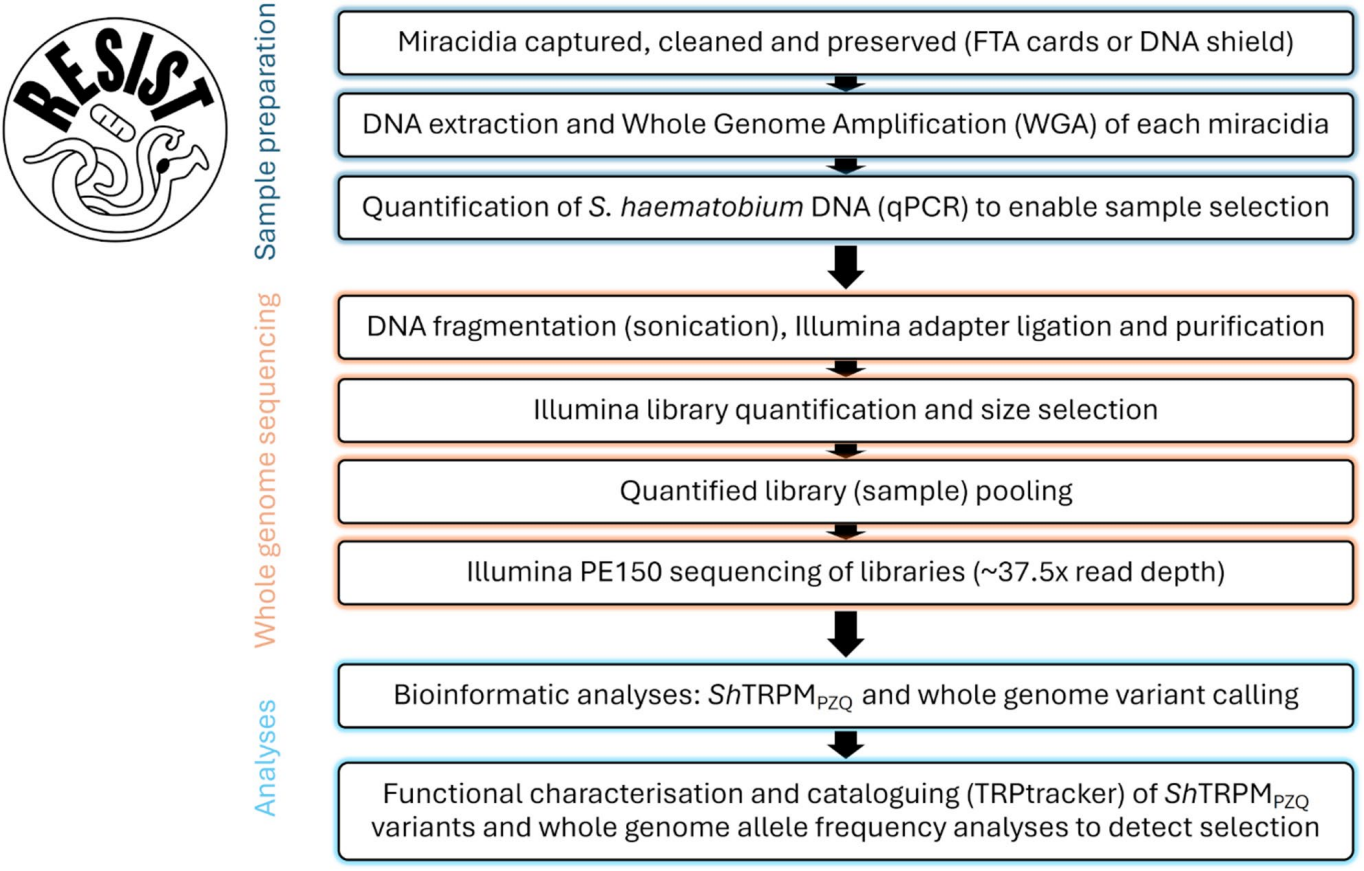
Workflow for the collection and genomic analyses of each individual *Schistosoma haematobium* miracidium and the RESIST logo

WGS data will be obtained from individual miracidia following the steps shown in Fig. 2. For each *S. haematobium* miracidium, WGS data will be aligned to the chromosome-level assembly of the *S. haematobium* reference genome (Shaem.V3) [32]. Variants, including Single Nucleotide Variants/Polymorphisms (SNVs/SNPs) and indels, observed across the genome will be investigated similarly to that previously described [31].

With *Sm*TRPM_PZQ_ now being recognised as the key genomic target for PZQ action, our primary analyses will focus on this gene’s *S. haematobium* ortholog (*Sh*TRPM_PZQ_), located on the *S. haematobium* chromosome three (TRPM2_3, GeneID = 24593960 or XM_051218923.1 [18]). Nucleotide variation and amino acid mutational frequency profiles of *Sh*TRPM_PZQ_ will be generated and compared to investigate the diversity of this gene across *S. haematobium* populations on Pemba and to determine variation across geographically isolated locations and between collection years. A portfolio of *Sh*TRPM_PZQ_ variants will be curated and added to the community-wide catalogue of TRPM_PZQ_ mutations available via www.trptracker.live/ [26]. Variants in the coding sequence of *Sh*TRPM_PZQ_ causing non-synonymous changes relative to the reference genome, will be functionally profiled using the established Ca^2+^ reporter assay [15, 26], prioritising those causing mutations within functional domains (e.g. TRPbox that may be critical for PZQ recognition by *Sh*TRPM_PZQ_). The mutant *Sh*TRPM_PZQ_ sensitivity to PZQ (EC_50_) and peak response (B_max_) will be measured relative to the wild type *Sh*TRPM_PZQ_, and shifts in the proteins ‘relative activity’ [26] will be stratified into the functional categories: ‘wild type’; ‘decreased PZQ sensitivity’; ‘sensitized’. Causative nucleotide polymorphisms in the relevant *S. haematobium* miracidia will also be validated by targeted PCR amplification of the polymorphic DNA region and Sanger sequencing.

The frequency and geographical distribution of *Sh*TRPM_PZQ_ mutations, having an association with reduced PZQ efficacy, will be used to model the occurrence and spread of PZQ-resistant genotypes. In particular, *Sh*TRPM_PZQ_ variants observed pre- and post-MDA (from the two schools that are part of the longitudinal studies) will be compared to identify any genetic signatures of positive selection of *S. haematobium* variants that may indicate PZQ resistance of adult *S. haematobium* worms within the human host. Additionally, WGS data from miracidia collected pre- and post-MDA from the same individuals will be analysed to identify relationships between the populations with a specific aim of identifying treatment failure *versus* maturation of juvenile worms present during treatment.

Both the *Sh*TRPM_PZQ_ variant and the WGS data will be analysed from the *S. haematobium* miracidia collected from the 17 schools to provide data on geographical genetic variation of the *S. haematobium* populations in relation to infection prevalence and intensity. Moreover, this dataset will be augmented with *Sh*TRPM_PZQ_ variant and WGS data generated from individual *S. haematobium* miracidia collected from these 17 schools in both 2012 and 2017 (collected as part of continuous schistosomiasis control activities in Zanzibar and curated with SCAN [21]).

### Management of data and biological material

#### Project data

All participant registration data collected by field enumerators in the 17 study schools will be recorded directly in ODK questionnaires using tablet computers (Samsung Galaxy Tab A 2019).

The results of laboratory examinations and PZQ MDA participation will be captured initially on paper forms, already containing each participant’s ID code. The results will be double-entered into an electronic database (Microsoft Excel 2016) already containing each participant’s ID code by two experienced data entry staff. The double-entered data will be cleaned and merged by the staff of Swiss TPH using the statistical software StataBE 19 (StataCorp, College Station, Texas, United States of America). Discrepancies in the data entries will be traced back to the original paper records at PHL-IdC and corrected. All versions of data entry, correction and cleaning will be labelled continuously and captured for data traceability.

Project data will be handled with discretion and will only be accessible to authorized personnel who require the data to fulfil their duties within the scope of this research project.

Within project-specific documents, participants are identified only by their unique participant ID code. Only coded samples will be subjected to parasitological and molecular examination. Of note, only the parasitological examination will reveal health-related data (*S. haematobium* infection status) of participants. The genetic analyses will target the parasite larvae (miracidia) isolated from the *S. haematobium* eggs, but no human biological material.

The names of participants derived through children’s registration in ODK will be separated from the remaining registration information and stored as a separate electronic file that matches the participants’ names and residencies with their personal ID code. The participant identification list for the longitudinal survey will be accessed, and a printed version will be used during MDA to capture participants’ treatment with PZQ and to call children from class for urine collections on Days 2–6 before and after MDA. This participant identification list and MDA information will then be used by the RESIST study team at PHL-IdC for data capturing.

When not in use, the paper forms will be stored in a safe locked cabinet in an office at PHL-IdC. The electronic versions of the participant identification list will be stored on a password protected computer at PHL-IdC and, password protected plus backed-up servers at Swiss TPH, NHM and Erasmus MC.

#### Biological material

The biological material is not identified by participant name but by a unique participant ID code.

After examination for *S. haematobium* infection and isolation of miracidia from urine samples containing *S. haematobium* eggs, excess urine from all samples collected during the cross-sectional surveys in 15 schools will be discarded. Excess urine from samples collected during the longitudinal surveys in two schools will be stored in 50 ml plastic tubes labelled with the participant ID code, in a -20 °C freezer at PHL-IdC for further research purposes.

The isolated miracidia will be stabilized on FTA cards (Qiagen, Hilden, Germany), which allow the sample to be stable at room temperature mitigating the risk imposed with the need for cold storage and/or with DNA/RNA Shield (Zymo Research, CA, USA) and stored at -20 °C. Fixed miracidia will be couriered securely to the NHM in London for genetic analyses.

### Statistics

#### Determination of sample size and power calculations

For the cross-sectional surveys in 15 schools, where only one pre-MDA urine sample will be collected per participant, we aim to collect urine samples from 1,200 participants per school to quantify genomic diversity of *S. haematobium*. Given that little information about genomic diversity of *S. haematobium* exists, we pragmatically chose to sample all attending children with a maximum target of ~ 1,200 children sampled per school, which is the maximum feasible to process within one working week per school and fits within the project budget.

For the longitudinal studies in two schools, where quintuple urine samples will be collected per participant before and after MDA, power calculations were done for non-inferiority and inferiority testing of PZQ efficacy. With the combination of non-inferiority and inferiority testing, a type I error can at most occur in one of the two tests and not for both at the same time: drug efficacy cannot both be equal to the expected efficacy and lower than the non-inferiority margin at the same time. Therefore, to arrive at an overall risk of a type I error of 0.05 for the two tests combined, we set al.pha to 0.10 for each of the two tests. Further, as Denwood et al. demonstrated [24], if a study is sufficiently powered for the non-inferiority testing, then it will automatically be sufficiently powered for the inferiority test as well. Therefore, the power calculations were done based on the non-inferiority test with alpha = 0.10 (effective type I error of 0.05 for the two tests combined) and a desired power of 90% (type II error of 0.10). Given the expected pre-MDA prevalence of egg-positivity of 5% in the two schools, and the typical highly overdispersed distribution of egg counts between individuals and within individuals (upon repeated testing over multiple days), we determined that quintuple testing of 500 individuals (over five days), both before and after PZQ treatment, would meet the desired maximum type I and II errors. The expected level of overdispersion of egg counts within and between individuals was based on previously quantified levels of variation in egg counts from intestinal parasitic worms (soil-transmitted helminths), as these measures have not been quantified before for *S. haematobium* (but will be as part of this project).

#### Datasets to be analysed and analysis populations

All data from participants who meet the eligibility criteria, provide consent, and provide a urine sample will be included in the analyses. Data will be analysed per school as well as across all schools combined, to assess variation in infection levels and genomic diversity of *S. haematobium* within and across schools. Assessment of *S. haematobium* genomic diversity will be based on individuals with detectable eggs in their urine and from whom at least one miracidium is successfully isolated and whole-genome sequenced. PZQ efficacy will be calculated based on only the individuals from the two schools where both pre- and post-MDA data are collected.

#### Statistical analyses

To address the study objectives that pertain to the cross-sectional surveys and longitudinal studies in 17 schools in Pemba (secondary objectives 1) and 2)), we will analyse data descriptively. We will determine the *S. haematobium* prevalence for each school and intensity of infection (*S. haematobium* egg counts / 10 ml urine) for each participant of the cross-sectional surveys conducted in 15 schools. In the two schools that were part of the longitudinal studies, we will assess the *S. haematobium* prevalence and intensity of infection before and after MDA. Since quintuple urine samples will be collected per participant before and after MDA, we will consider participants as *S. haematobium*-infected if at least one *S. haematobium* egg is detected in at least one of their urine samples before or after MDA, respectively. Infection intensity of each participant will be calculated as the mean egg count from all of their available samples before and after MDA, respectively.

To address the study objectives that pertain to the genetic analyses of *S. haematobium* miracidia from the different locations in Pemba, to identify mutations that may relate to PZQ resistance (primary objective and secondary objectives 3)-9)), we will perform functional annotation of proteins from variants using a custom database generated using SnpEff [33], and analyse mutational frequencies in the *Sh*TRPM_PZQ_ region in R (https://www.r-project.org/). We will assess genome-wide population structure using the pairwise fixation index (F_ST_) calculated in non-overlapping, sliding windows of the genome using an unbiased F_ST_ measure *pixy* [34], allowing for pairwise comparisons of populations partitioned by location (e.g., school) or by timepoints (e.g. 2012 *versus* 2025). In parallel, we will perform population stratification analysis using principal components calculated in PLINK 2.0 (www.cog-genomics.org/plink/2.0/) [35] and determine the number of ancestral (i.e. genetically distinct) populations in ADMIXTURE [36].

To assess the genetic profile of *S. haematobium* miracidia collected before and after MDA from two different schools in Pemba, we will do pairwise comparisons of parasite populations based on F_ST_, partitioned by the individual-level egg reduction rates. Next, we will test whether any change in allele frequency at the *Sh*TRPM_PZQ_ locus between pre- and post-MDA samples differs from the genome wide allele frequency changes. To perform hypothesis testing on this, we will identify a set of ~ 100 “control” SNPs that are outside of the *Sh*TRPM_PZQ_ gene that display comparable pre-MDA allele frequencies to those at the *Sh*TRPM_PZQ_ locus. Pre- and post-MDA allele frequencies will then be calculated for each SNP in *Sh*TRPM_PZQ_ and control loci. The null hypothesis is that the change in allele frequencies across *Sh*TRPM_PZQ_ will not be significantly different to the allele frequency changes across the control loci. Rejection of the null hypothesis would indicate that one or more alleles at *Sh*TRPM_PZQ_ changed in frequency significantly more than in the control loci, which would be consistent with a locus-specific selection.

To assess the association between presence of *Sh*TRPM_PZQ_ SNPs and within-individual changes in egg counts pre- and post-MDA, we will use a bespoke statistical model implemented in a Bayesian framework that accounts for variation in egg counts between and within individuals, implemented in the probabilistic programming language Stan.

Other statistics will be used to identify evidence of selection across the genomes, including the alpha statistic calculated using Bayescan v2.1 and identification of regions of extended homozygosity (xpEHH) using the rehh v2.0.2 R package [37, 38]. Other packages and statistics may be applied to the schistosome genome data as appropriate.

To address the study objective that pertains to PZQ efficacy (secondary objective 10), we will measure PZQ efficacy in terms of reductions in egg counts (ERR) post-MDA. With the egg count data of the two schools where samples will be collected pre- and post-MDA, we will perform two linked types of hypothesis testing to classify PZQ efficacy at the school level: inferiority and non-inferiority testing of PZQ efficacy compared to the expected efficacy of 98% and a non-inferiority margin of 5%-points, following the statistical framework by Denwood et al. [24]. For the non-inferiority test, the null hypothesis is that PZQ efficacy is equal to the expected effect minus the non-inferiority margin (98% – 5% = 93%). The alternative hypothesis is that PZQ efficacy is at least 93%. For the inferiority test, the null hypothesis is that PZQ efficacy is equal to the expected efficacy (98%), and the alternative hypothesis is that PZQ efficacy is less than 98%. If the null hypothesis for the non-inferiority test is rejected, PZQ efficacy will be considered “adequate”, even if the null hypothesis for the inferiority test is rejected. If the null hypothesis for the non-inferiority test cannot be rejected and the null hypothesis for the inferiority is rejected, PZQ efficacy will be considered “reduced”. If neither of the two null hypotheses can be rejected, PZQ efficacy will be considered “inconclusive”. In contrast to Denwood et al., we will adopt a general linear mixed modelling framework so that we can include all individuals in the analysis, even those with missing data for some of the five repeated measures (pre- or post-MDA).

Study objectives that relate to mathematical modelling (secondary objectives 11 and 12) are addressed in the “Mathematical modelling” section.

#### Handling of missing data

We will not replace individual participants who drop out with new participants. For the 15 schools where only a single pre-MDA urine sample per subject is collected, we will analyse data only from individuals who provide a urine sample of at least 10 ml. In the two schools where we aim to collect five samples over five different days before PZQ treatment and five samples over five days post-MDA, some individuals may not provide samples for all of the sampling time points. These individuals will still be included in the analysis as their data provide potentially valuable information about the distribution of egg counts within the population. The missingness of those data will be considered “at random”, not related with the outcome of interest (egg counts or reduction thereof), which will allow us to incorporate the non-missing data for those individuals in a generalized linear mixed modelling framework.

### Mathematical modelling

#### Mathematical modelling of population dynamics

To predict trends in infection and efficacy of PZQ against *S. haematobium* under current and potential future control scenarios, we will adapt an existing individual-based transmission model for drug resistance in soil-transmitted helminths [14]. We will update this model to capture *S. haematobium* biology, including adult worm lifespan and mating processes, limiting mechanisms in transmission (density-dependent worm fecundity, host immunity-dependent parasite establishment and drug efficacy, and transmission to snails), clonal production of cercariae in the snail host, lack of PZQ efficacy against juvenile worms, and age patterns in (re-) infection. Based on literature on experimentally induced resistance in *Schistosoma* lab strains, we will assume that resistance is monogenic and recessive [15–17]. The model will be calibrated to reproduce the geographical and temporal patterns in infection from historical and newly collected data. “Baseline” genetic variation in the simulated population will be set to represent the earliest observed genetic diversity. Using data on the history of control as model inputs, we will predict trends in infection (egg prevalence and intensity), genetic diversity (allele, haplotype, and genotype frequencies), and drug efficacy (ERR) up to 2017 and 2025 and compare predictions to data from those time points. We assume that drug efficacy (ERR) against non-resistant parasites is 98% with proper dosing and absorption [39]. For resistant parasites, we will assume a 90%, 95%, or 99% reduction in the probability of PZQ killing worms, as observed in other flatworms such as *Fasciola* spp [19, 20]. This assumption will be refined after functional profiling data from genetic variants become available, combined with the previously observed correlation between functional profiles and clinical efficacy of PZQ against flatworms [19, 20]. If no reduced efficacy or resistance-conferring alleles are detected, we will broaden the range of phenotypes down to a 50% reduction in efficacy to determine whether such degrees of resistance can evade detection and to what extent they are of concern for control targets.

Next, we will predict the impact of various alternative future scenarios for “standard” MDA:

- 6-monthly, annual, or every two or three years;
- School- or community-based;
- Optionally with treatment of pre-school age children with a paediatric PZQ formulation.

We will further consider the following mitigation strategies:

- Multi-drug treatment with PZQ and a hypothetical second drug from a different family (i.e., not affected by resistance), considering a range of potential efficacies of this second drug against adult and/or juvenile worms [13];
- Rotating drug treatment with PZQ and the aforementioned hypothetical second drug;
- Test-and-treat: targeted treatment of heavily infected individuals with PZQ, based on individual-level diagnosis;
- Improved access to water, sanitation and hygiene [40].

We will evaluate these strategies in terms of prospects of sustained control (i.e., maintaining prevalence of heavy infection < 1%), interruption of transmission, and trends in drug efficacy.

#### Design cost-efficient survey strategies for pharmacovigilance

To inform future pharmacovigilance efforts, we will design cost-efficient survey strategies to evaluate drug efficacy based on ERRs, using a recently developed Monte Carlo simulation framework for soil-transmitted helminths [23]. We will adapt and re-quantify this framework with regard to the following aspects of *S. haematobium*, based on the following data:

- Natural variability of *S. haematobium* egg counts within and between individuals, based on quintuple egg count data from two schools;
- Between-school variation in infection levels, based on the egg count data from all 17 schools;
- Operational cost of drug efficacy surveys (e.g., personnel, consumables, transport) as previously estimated [41, 42] and updated based on project expenses.

With this framework, we will identify survey designs (number of individuals per school, number of urine samples per individual) that provide the desired power (80% or 90%) for the detection of reduced efficacy at the lowest cost. Power will be calculated based on a recently published statistical framework for the classification of drug efficacy in veterinary parasites [43].

### Timeline

Overall, the RESIST project will be implemented over three years, from September 2024 to August 2027. The longitudinal study will be conducted in two schools in Pemba from November 2024 to March 2025. MDA in these two schools will be aligned with the RESIST project and conducted in November 2024 in the first school and in February 2025 in the second school, respectively. The cross-sectional surveys in 15 schools will be implemented from May to July 2025. Each school will receive MDA immediately following the survey. Genomic analyses of miracidia collected from *S. haematobium*-positive individuals will be done at NHM in London between February 2026 and May 2027. Statistical analyses and mathematical modelling will be done at Erasmus MC in Rotterdam between December 2024 and August 2027.

### Dissemination of findings

Throughout the project, the RESIST study team will report to the Zanzibar Ministry of Health, district authorities, shehas, and teachers regarding the project objectives, findings and outcomes. For this purpose, annual face-to-face meetings will be conducted in Unguja and Pemba at the start of the project, once the parasitological surveys in schools are concluded and at the end of the project.

Moreover, findings will be discussed regularly in online meetings with stakeholders from the RESIST Project Advisory Board, World Health Organization, Expanded Special Project for Elimination of Neglected Tropical Diseases (ESPEN), Merck, Global Schistosomiasis Alliance, NTD Unit of the Zanzibar Ministry of Health, implementing partners in PZQ roll out and the Wellcome Trust.

Finally, all data and project outputs will be disseminated to the wider scientific community through open-access publications, presentations at international conferences, policy briefs, and press releases. Immediately upon completion, all parasitological and genetic data, as well as model source code will be made available in open-access platforms.

If data show that PZQ resistant *S. haematobium* populations exist within our study sites, we will inform involved stakeholders and discuss how to address the findings, given that currently, there is a lack of alternative drugs or drug combinations.

To the communities in Zanzibar, the information will be disseminated in a way that does not cause alarm. Focus will remain on the observed successes of PZQ treatment, particularly in relation to morbidity control and the reduction in transmission, and even transmission interruption in some areas of Zanzibar. Discussion will focus on the need for communities to try and reduce transmission by the use of safe water sources and sanitation, whilst also continuing to receive PZQ treatment due to the known positive clinical outcomes observed for infected individuals.

## Discussion

After two decades of regular MDA with PZQ, the persistence of *S. haematobium* hotspots is impeding the progress towards elimination of schistosomiasis on the Zanzibar archipelago. If the intensive use of PZQ has led to selection of drug-resistant *S. haematobium* populations, it will be essential to understand how this resistance may spread and what can be done to mitigate its impact on transmission control. Building on the latest insights of the molecular basis of PZQ action and resistance in schistosomes, the RESIST project will deliver the first-ever genetic characterisation of the molecular target of PZQ (TRPM_PZQ_) in a natural *S. haematobium* population on Pemba. Our study will determine whether PZQ efficacy is reduced in hotspot areas and, if so, whether this is linked to genetic variants in *S. haematobium*. By integrating the collected epidemiological, genetic, and PZQ efficacy data in mathematical models, we will generate evidence-based recommendations on resistance mitigation strategies and cost-effective pharmacovigilance of PZQ.

We foresee the following practical and operational challenges involved in the different parts of the RESIST project. First, with regard to implementing the cross-sectional surveys and longitudinal studies and MDA in a total of 17 schools over a period of almost one year, there is a need to closely collaborate with the Ministry of Education and the Ministry of Health, as well as with school principals, teachers, parents and students. Our surveys will have to be adapted and aligned with school curricula, examination periods, public holidays and school holidays. In particular, for the two schools and students participating in the longitudinal studies, where participants are invited to submit five urine samples before and after MDA, respectively, a certain time commitment as well as endurance in submitting several samples is involved. To ensure the consent of teachers and parents and assent and compliance of students, we will have several meetings with representatives of the Ministries, school principals and teachers, a parent delegation and the students themselves to explain the procedures, purpose and importance of the study. In all surveys, to minimise disruption to teaching, we will facilitate the collection of urine samples and ensure a time-saving procedure by adapting our work to the morning and afternoon shifts in the schools, collecting samples per class with trained staff from PHL-IdC and using collection containers pre-labelled with stickers clearly showing the participant ID code for each child on cup and lid. To ensure accurate examination of the urine samples, in the laboratory at PHL-IdC, experienced and well-trained technicians will organize the sample flow, conduct the examination with urine filtration microscopy, collect miracidia and record results on CRFs pre-labelled with the participant ID code for each child. Regarding the PZQ MDA in each school, we will closely collaborate with the NTD Unit of the Zanzibar Ministry of Health. The MDA will be implemented by the RESIST study team and schoolteachers, supervised by qualified staff of the NTD Unit. The timing of the MDA will be agreed with the manager of the NTD Unit as well as with school principals.

Second, with regard to miracidia collection and genomic analyses, our methodology poses several challenges. Since urine samples of small volumes (e.g. 10 ml) leave no residual urine for miracidial hatching (once the ~ 10 ml is used for urine filtration microscopy), participants will be instructed on the importance of filling the 100 ml containers, if possible. Moreover, whole genome sequencing of individual miracidia requires transfer of miracidia one-by-one, from the hatching dish to preservation media. Transfer failures or storage degradation will only become apparent after DNA extraction and WGA. To reduce these losses, we have prioritized front-line laboratory staff training in miracidia transfer and the downstream technical steps (DNA isolation, WGA and quantitative PCR), both to support possible transfer of these steps to the PHL-IdC, and to emphasise the importance of early-stage collection.

Our analyses will include comparisons between defined populations of miracidia, whether that be comparing populations between separate individual participants between populations collected from different schools or between different sampling points from the same individual participant (i.e. pre- and post-MDA). The number of miracidia available in each population (per individual, per school, or per pre- and post-MDA group) will inevitably differ due to natural variation in infection intensities (i.e. fewer miracidia will be available from individuals with fewer worms), and in particular in post-MDA samples, which we predict to yield fewer miracidia due to expected high cure rates associated with PZQ treatment. Although such disparities in miracidia sample sizes are unavoidable, we will mitigate potential sample-size bias between miracidia populations by applying genetic and statistical methods designed to accommodate for unequal sampling depth. To reduce sample dropout during DNA preparation for WGS, we will optimise the DNA isolation and WGA protocols using previously collected material. Finally, to manage the > 1000 genomic variant call files, we will use the Genome Analysis Tool Kit [44] GenomicsDB datastore format, which provides efficient consolidation and iterative addition of new samples. Our WGS approach is a compromise, since a targeted sequencing approach would be more efficient if we were focused solely on a single locus (i.e. *Sh*TRPM_PZQ_). However, we have adopted a whole genome approach to maximise the utility of our dataset, since drug efficacy may be influenced by gene products encoded by other loci.

Third, regarding modelling, a main challenge concerns the dynamics of *S. haematobium* transmission and evolution of drug resistance. Here, we will adapt our analyses to deal with uncertainties about the complexity of schistosome biology and the associated host-parasite interactions. Examples of such uncertainties are the role of acquired host immunity on parasite establishment, adult worm mating (adult worms are likely not completely monogamous), and the mechanism(s) that ensure that infection levels in snails are consistently low, regardless of infection levels in humans. By means of scenario and sensitivity analyses, we will investigate which uncertainties matter most and thus deserve more attention in future research, and which do not matter as much in light of monitoring and mitigation of drug resistance, and may therefore be simplified or disregarded in the model. Of note, even if no evidence of resistance or genetic selection is found, modelling will make a useful contribution to our understanding of the population dynamics of drug resistance by using literature-based assumptions about the link between genotypes and phenotypes, for instance, based on the data on experimentally induced resistance in schistosomes. Although this will not be direct evidence, but merely “speculative”, it will provide useful insights into potential (worst-case) scenarios for the population dynamics of drug resistance in *S. haematobium*. This will help generate (i) further hypotheses that can be tested in future research, and (ii) recommendations for strategies to monitor emergence of potential drug resistance.

In summary, the RESIST project will have important impacts: it will identify if PZQ resistance is an endgame challenge for *S. haematobium* and how it may be monitored (e.g., diagnostic genotyping assays and parasitological pharmacovigilance strategies) and mitigated (e.g. targeted control interventions). Our findings will have highly relevant implications for novel drugs in the pipeline [13], informing what the minimum required efficacy of such novel drugs would need to be to mitigate the impact of potential PZQ resistance. This study will also provide valuable data on infection prevalences and intensity of *S. haematobium* infections in 17 school across Pemba, supporting the ongoing assessment of urogenital schistosomiasis control in the Zanzibar archipelago. We also envisage that the data will highlight the critical need for more intensive multi-day parasitological surveys to obtain a more accurate estimate of infection levels in schistosomiasis elimination settings, so that appropriate and timely interventions (including MDA) can be implemented. Our study will hence advance countries further towards elimination and beyond. Last and importantly, we envisage that this project will inform the design of a future Africa-wide multi-country study to assess the presence and implications of potential PZQ resistance in schistosomiasis-endemic areas.

## Data Availability

Upon termination of the sample and data analyses for this project, all data will be anonymized. Anonymized data will be curated and stored in the data repository “Infectious Diseases Data Observatory” (IDDO; https://www.iddo.org/) for an unlimited period of time. Miracidia samples not used for genetic profiling in this project will be curated in the Schistosomiasis Collection at the Natural History Museum (SCAN; https://scan.myspecies.info/) for an unlimited time and will be made available for further research upon permission of PHL-IdC.All WGS data from each *S. haematobium* miracidia analysed will be deposited and curated within open-access genetic resources (NCBI SRA; https://www.ncbi.nlm.nih.gov and https://www.trptracker.live/). All model source codes, documentation, and codes to perform analyses for this project will be version-controlled and made publicly available via GitLab (https://gitlab.com/), and model-simulated data will be made available via Zenodo (https://zenodo.org/).

## Abbreviations

CRF: Case report form
EKNZ: Ethikkommission Nordwest- und Zentralschweiz
ERR: Egg reduction rate
DNA: Deoxyribonucleic acid
F_ST_: Fixation index
ICF: Informed consent form
IDDO: Infectious Diseases Data Observatory
MDA: Mass drug administration
NHM: Natural History Museum
NTD: Neglected tropical diseases
PCR: Polymerase chain reaction
PHL-IdC: Public Health Laboratory-Ivo de Carneri
PZQ: Praziquantel
SCAN: Schistosomiasis Collection at the Natural History Museum
SNP: Single nucleotide polymorphism
SNV: Single nucleotide variant
Swiss TPH: Swiss Tropical and Public Health Institute
TRPM_PZQ_: Transient Receptor Potential Ion Channel for PZQ action
WGA: Whole genome amplification
WGS: Whole genome sequencing
WHO: World Health Organization
ZAHRI: Zanzibar Health Research Institute
ZEST: Zanzibar Elimination of Schistosomiasis Transmission
